# Comparison of Procalcitonin, C-reactive Protein, and Interleukin-6 in the Postoperative Course After Lung Decortication Surgery: A Prospective Observational Study

**DOI:** 10.7759/cureus.95022

**Published:** 2025-10-21

**Authors:** Vipin K Singh, Sankara Narayanan, Shefali Gautam, Monica Kohli

**Affiliations:** 1 Anaesthesiology, King George's Medical University, Lucknow, IND

**Keywords:** biomarkers, c-reactive protein, interleukin -6, lung decortication, pleural empyema, postoperative sepsis, procalcitonin

## Abstract

Introduction

Lung decortication is a surgical intervention performed to remove the fibrous pleural layer in conditions like pleural empyema or chronic pleuritis. Despite its therapeutic benefits, the postoperative period poses a high risk for complications such as infections and sepsis, which can significantly affect recovery. Biomarkers such as interleukin-6 (IL-6), C-reactive protein (CRP), and procalcitonin (PCT) are increasingly employed to aid early detection of such complications.

Objectives

To evaluate and compare the roles of IL-6, CRP, and PCT in detecting postoperative sepsis in patients undergoing lung decortication and to determine their clinical utility in guiding early management decisions.

Methods

This observational study was conducted in the Departments of Anaesthesiology and General Surgery, King George’s Medical University, Lucknow. A total of 112 patients aged 18-65 years with clinically proven pleural empyema undergoing elective lung decortication were enrolled. Patients on preoperative anti-inflammatory or immunosuppressive drugs were excluded. Preoperatively and on postoperative days 1, 3, and 5, biomarkers were analyzed. Data analysis was performed using SPSS 26.0 (IBM Corp., Armonk, NY, US), applying t-tests, chi-square tests, and analysis of variance (ANOVA) with a significance threshold of p<0.05.

Results

Of 112 patients, 44 (39.3%) developed sepsis. Septic patients were older (mean age 40.84±14.08 years) than non-septic patients (mean age 28.09±8.00 years). PCT levels were significantly higher in septic patients across all time points (p<0.001), declining over time postoperatively. CRP levels were significantly elevated in septic patients on days 3 and 5. IL-6 and CRP showed limited but supportive diagnostic value. Mortality was higher in the septic group (6.8%), though not statistically significant.

Conclusion

Procalcitonin is the most reliable biomarker for the early detection and monitoring of postoperative sepsis following lung decortication. Combining PCT with CRP and IL-6 enhances diagnostic accuracy and informs timely therapeutic interventions.

## Introduction

Postoperative sepsis remains a major determinant of morbidity, prolonged hospitalization, and mortality in thoracic surgical patients [1]. Despite advances in perioperative management, differentiating between the physiological inflammatory response and infection-related sepsis continues to be a diagnostic challenge during the early postoperative phase [2]. Conventional clinical markers, such as temperature, leukocyte count, and C-reactive protein (CRP), lack specificity and often fail to distinguish infectious from non-infectious inflammation [3].

Procalcitonin (PCT) and interleukin-6 (IL-6) have gained attention as early indicators of bacterial infection and systemic inflammation [4]. PCT, a pro-peptide of calcitonin produced mainly by parenchymal tissues during bacterial endotoxin exposure, rises rapidly within 6-12 hours of infection onset and correlates with sepsis severity [5]. In contrast, CRP peaks after 24-48 hours and may remain elevated in sterile postoperative inflammation [6]. IL-6, a pleiotropic cytokine released by activated macrophages and endothelial cells, increases sharply in the early inflammatory phase but can also be induced by surgical trauma and tissue manipulation [7].

Lung decortication surgery involves extensive pleural dissection and manipulation of inflamed tissues, which inherently generate a robust inflammatory response [8]. This makes early differentiation between infection-related sepsis and sterile inflammatory changes particularly difficult. Previous studies have suggested that dynamic assessment of biomarkers rather than isolated values may enhance diagnostic accuracy [9]. However, comparative data on PCT, CRP, and IL-6 in patients undergoing lung decortication are limited.

Therefore, the present study was designed to compare the temporal trends and diagnostic accuracy of PCT, CRP, and IL-6 in detecting early postoperative sepsis following lung decortication. By analyzing biomarker kinetics and receiver operating characteristic (ROC) parameters, this investigation aims to identify the most reliable early predictor of sepsis and facilitate timely clinical intervention.

## Materials and methods

Study design and setting

This was a prospective, observational study conducted at a single tertiary-care center over an 18-month period. The study was designed to evaluate and compare the diagnostic accuracy of three inflammatory biomarkers--PCT, CRP, and IL-6--for the early detection of postoperative sepsis following lung decortication surgery. The research protocol adhered to the principles of the Declaration of Helsinki, and approval was obtained from the Institutional Ethics Committee prior to patient enrolment. Written informed consent was obtained from all participants before inclusion.

Study population

Patients aged 18-70 years who were scheduled to undergo lung decortication for chronic empyema or fibrothorax under general anesthesia were prospectively recruited. Inclusion criteria comprised patients with radiologically and clinically confirmed trapped lung who were hemodynamically stable and fit for major thoracic surgery. Exclusion criteria included pre-existing systemic infection, chronic renal or hepatic failure, immunosuppressive therapy, autoimmune disorders, pregnancy, or refusal to consent.

A total of 112 patients fulfilling the eligibility criteria were enrolled consecutively. Patients were followed from the preoperative period until postoperative day (POD) 5. Based on postoperative course and clinical findings, the patients were categorized into septic and non-septic groups using predefined diagnostic criteria.

Definition of postoperative sepsis

Sepsis was defined according to the Third International Consensus Definitions (Sepsis-3) as life-threatening organ dysfunction caused by a dysregulated host response to infection [1]. For the purpose of this study, postoperative sepsis was diagnosed when the patient exhibited both: clinical evidence of infection (fever > 38 °C, tachycardia > 100 bpm, respiratory rate > 20/min, or purulent drainage from the pleural space), and laboratory or radiologic confirmation (positive pleural/pus culture, new or persistent leukocytosis > 12 000/µL, or new infiltrates on chest radiograph).

Patients meeting these criteria within five postoperative days were classified as septic, while those without such findings were designated non-septic.

Sample collection and biomarker measurement

Venous blood samples were collected at four time points: Preoperative baseline (D0); Postoperative day 1 (POD 1); Postoperative day 3 (POD 3); Postoperative day 5 (POD 5).

Each sample (5 mL) was centrifuged at 3,000 rpm for 10 minutes, and the serum was separated and stored at −20 °C until analysis. PCT levels were measured using a quantitative enzyme-linked fluorescent assay (ELFA) based on the sandwich immunoassay principle. CRP concentrations were determined by an immunoturbidimetric method using an automated chemistry analyzer. IL-6 was quantified using a solid-phase enzyme-linked immunosorbent assay (ELISA). All assays were performed in duplicate by the same technician to minimize inter-assay variability. The results were expressed in ng/mL for PCT, mg/L for CRP, and pg/mL for IL-6.

Clinical monitoring and data collection

All patients received standardized perioperative care, including broad-spectrum antibiotic prophylaxis, thoracic epidural analgesia, and postoperative chest-tube drainage. Clinical parameters, such as heart rate, blood pressure, respiratory rate, oxygen saturation, and temperature, were recorded every four hours. Laboratory investigations, including total leukocyte count (TLC), differential count, serum creatinine, and chest-radiograph findings, were documented daily until POD 5.

Demographic data (age, sex), comorbidities, duration of symptoms, indication for surgery, and operative details were recorded in a predesigned case record form. The occurrence of postoperative complications, particularly sepsis and wound infection, was noted.

Statistical analysis

Data were entered into Microsoft Excel (Microsoft Corporation, Redmond, WA, US) and analyzed using IBM SPSS Statistics version 26.0 (IBM Corp., Armonk, NY, USA). Continuous variables were checked for normality using the Shapiro-Wilk test. Normally distributed variables were expressed as mean ± standard deviation (SD) and compared between groups using Welch’s independent t-test. Non-normally distributed variables were summarized as median (interquartile range) and analyzed using the Mann-Whitney U test. Categorical data were presented as frequencies and percentages and compared using the chi-square test or Fisher’s exact test where appropriate.

Serial changes in biomarker levels (PCT, CRP, IL-6) across postoperative days were analyzed using repeated-measures ANOVA or the Friedman test, depending on distribution characteristics. Correlations among biomarkers were assessed using Pearson’s or Spearman’s correlation coefficients.

To evaluate diagnostic performance, receiver operating characteristic (ROC) curves were constructed for each biomarker on POD 1. The area under the curve (AUC) with 95% confidence interval (CI) was computed by the bootstrap method (n = 1,000). The optimal cutoff value for each biomarker was derived using Youden’s index (J = Sensitivity + Specificity − 1). Diagnostic accuracy was expressed in terms of sensitivity, specificity, positive predictive value (PPV), negative predictive value (NPV), and Youden’s J. A p-value < 0.05 was considered statistically significant.

Ethical considerations

The study protocol was reviewed and approved by the Institutional Ethics Committee of King George's Medical University, U.P., prior to initiation. All participants provided written informed consent before enrollment. Confidentiality was maintained throughout data handling and analysis, and no identifiable personal information was disclosed in the study report.

## Results

Patient demographics and baseline characteristics

**Figure 1:**
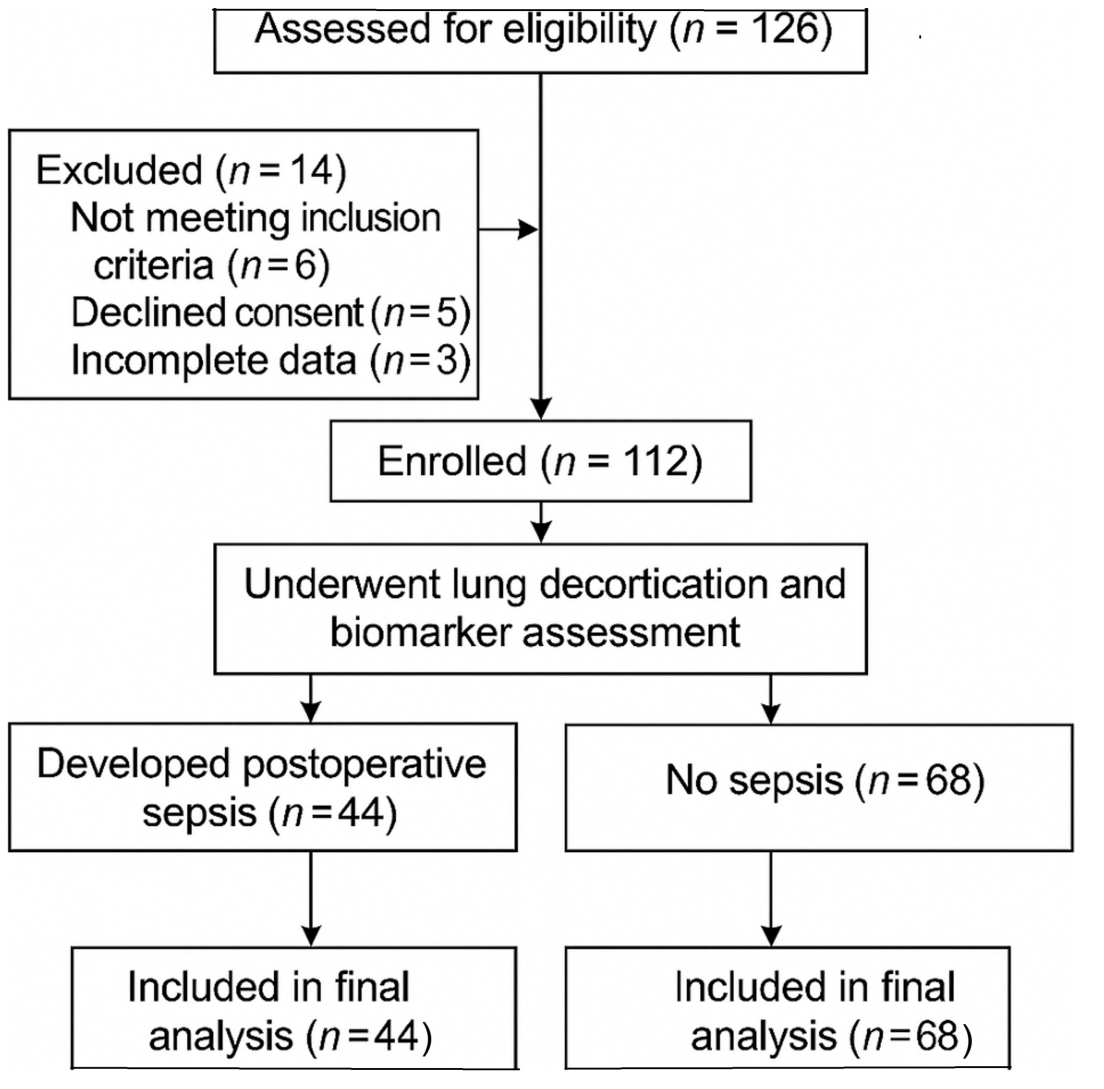
CONSORT flow diagram depicting patient enrollment, exclusions, biomarker sampling, and final analysis for the study on postoperative sepsis after lung decortication surgery CONSORT: Consolidated Standards of Reporting Trials

A total of 112 patients undergoing lung decortication were enrolled in the study (Figure 1).

The mean age of the cohort was 33.10 ± 12.43 years, with a male predominance (85.7%, n = 96). The mean baseline total leukocyte count (TLC) was 13,450 ± 6,800 cells/µL. No significant demographic differences were noted between the septic and non-septic groups at baseline (Table 1).

**Table 1:** Demographic and baseline characteristics TLC: total leukocyte count Note: Continuous variables are expressed as mean ± SD; categorical variables as n (%).

Characteristic	Overall
Total patients, n	112
Age (years), mean ± SD	33.10 ± 12.43
Sex, n (%)	Male: 96 (85.7%); Female: 16 (14.3%)
Baseline TLC (cells/µL), mean ± SD	13 450 ± 6 800

Postoperative incidence of sepsis

Out of 112 patients, 44 (39.3%) developed postoperative sepsis within 5 days following surgery, whereas 68 (60.7%) remained non-septic. The septic group exhibited higher baseline inflammatory indices and more pronounced postoperative biomarker elevations.

Temporal trends of biomarkers

Table 2 summarizes the sequential variations of PCT, CRP, and IL-6 across four time points (preoperative, POD 1, 3, and 5). All three biomarkers showed a significant postoperative rise peaking on POD 1, followed by a gradual decline toward baseline by POD 5. Among them, PCT exhibited the most distinct kinetic profile-rising sharply on POD 1 and declining consistently thereafter, while CRP demonstrated a delayed reduction, and IL-6 showed greater inter-individual variability.

**Table 2:** Biomarker levels at each timepoint (descriptive summary) PCT: procalcitonin; CRP: C-reactive protein; IL-6: interleukin-6; POD: postoperative day Note: Mean ± SD shown for normally distributed variables and Median (IQR 25th–75th percentile) for non-normally distributed variables based on the Shapiro–Wilk test.

Biomarker (timepoint)	Value
PCT – Baseline	0.30 ± 0.16
PCT – POD 1	5.35 ± 3.86
PCT – POD 3	3.51 ± 2.13
PCT – POD 5	2.46 ± 1.01
CRP – Baseline	273.93 ± 50.82
CRP – POD 1	371.16 ± 72.85
CRP – POD 3	251.33 ± 33.94
CRP – POD 5	139.05 ± 24.22
IL-6 – Baseline	10.74 ± 3.29
IL-6 – POD 1	423.05 ± 201.31
IL-6 – POD 3	243.25 ± 135.08
IL-6 – POD 5	3.29 ± 3.35

Comparison between the septic and non-septic groups

As shown in Table 3, patients with postoperative sepsis had significantly higher mean values of PCT, CRP, and IL-6 at all postoperative time points (p < 0.05). The difference was most pronounced for PCT, where mean POD 1 levels were 5.35 ± 3.86 ng/mL in septic patients versus 1.52 ± 0.19 ng/mL in non-septic individuals (p < 0.0001). CRP and IL-6 also demonstrated significant but less discriminative separation between the two groups.

**Table 3:** Comparison between septic and non-septic patients PCT: procalcitonin; CRP: C-reactive protein; IL-6: interleukin-6; TLC = total leukocyte count; POD: postoperative day Note: Continuous data are presented as mean ± SD (for normally distributed variables) or median (IQR: 25th–75th percentile) for non-normal data, based on the Shapiro–Wilk test. p-values were computed using Welch’s t-test or the Mann–Whitney U test as appropriate; categorical data were analyzed using the chi-square test.

Variable	Septic (mean ± SD / n %)	Non-septic (mean ± SD / n %)	p-value
Age (years)	40.84 ± 14.08	28.09 ± 8.00	<0.0001
Sex, n (%)	Male 36 (81.8%); Female 8 (18.2%)	Male 60 (88.2%); Female 8 (11.8%)	0.4380
TLC (cells/µL)	18 437 ± 2 420	7 480 ± 2 655	<0.0001
PCT (ng/mL) – Pre-op	0.30 ± 0.16	0.14 ± 0.13	<0.0001
PCT (ng/mL) – POD 1	5.35 ± 3.86	1.52 ± 0.19	<0.0001
PCT (ng/mL) – POD 3	3.51 ± 2.13	1.00 ± 0.14	<0.0001
PCT (ng/mL) – POD 5	2.46 ± 1.01	0.51 ± 0.15	<0.0001
CRP (mg/L) – Pre-op	273.93 ± 50.82	260.44 ± 60.81	0.2250
CRP (mg/L) – POD 1	371.16 ± 72.85	350.50 ± 37.96	0.0520
CRP (mg/L) – POD 3	251.33 ± 33.94	228.82 ± 28.54	0.0350
CRP (mg/L) – POD 5	139.05 ± 24.22	129.51 ± 19.44	0.0210
IL-6 (pg/mL) – Pre-op	10.74 ± 3.29	10.30 ± 3.66	0.5190
IL-6 (pg/mL) – POD 1	423.05 ± 201.31	356.43 ± 130.09	0.0540
IL-6 (pg/mL) – POD 3	243.25 ± 135.08	198.69 ± 125.82	0.0830
IL-6 (pg/mL) – POD 5	3.29 ± 3.35	7.40 ± 3.33	0.0880

Correlation among baseline biomarkers

At baseline, weak positive correlations were observed among PCT, CRP, and IL-6 concentrations (Table 4). The highest correlation was found between CRP and IL-6 (r = 0.267), whereas the relationships between PCT and the other biomarkers were minimal (r < 0.20), suggesting that each marker reflects a distinct aspect of the inflammatory response.

**Table 4:** Pearson correlation matrix among baseline biomarkers PCT: procalcitonin; CRP: C-reactive protein; IL-6: interleukin-6 Note: Pearson’s correlation coefficients (r) used to assess linear relationships among baseline biomarker concentrations. All correlations are weak and non-significant (p > 0.05).

Biomarker	PCT (ng/mL)	CRP (mg/L)	IL-6 (pg/mL)
PCT (ng/mL)	1.000	0.174	0.152
CRP (mg/L)	0.174	1.000	0.267
IL-6 (pg/mL)	0.152	0.267	1.000

Diagnostic accuracy analysis (POD 1)

**Figure 2:**
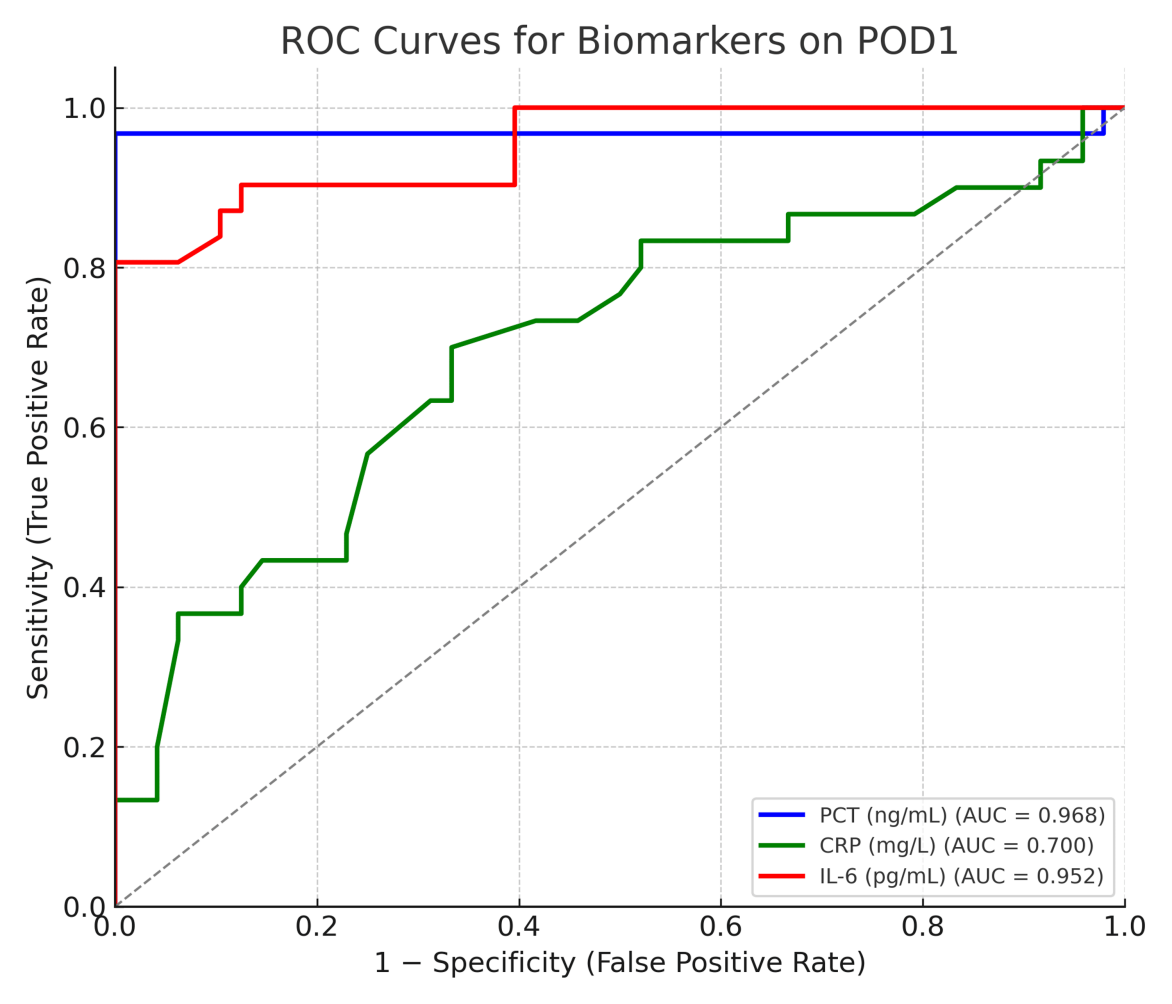
Receiver operating characteristic (ROC) curves for PCT, CRP, and IL-6 on POD 1 showing comparative diagnostic accuracy PCT: procalcitonin; CRP: C-reactive protein; IL-6: interleukin-6; POD: postoperative day

ROC analysis was performed for all three biomarkers on POD 1 to evaluate their predictive accuracy for postoperative sepsis (Table 5; Figure 2).

**Table 5:** Diagnostic accuracy of biomarkers on postoperative day 1 (POD 1) PCT: procalcitonin; CRP: C-reactive protein; IL-6: interleukin-6; POD: postoperative day; PPV: positive predictive value; NPV: negative predictive value Note: Optimal cutoffs identified by maximizing Youden’s J statistic (J = Sensitivity + Specificity − 1). Diagnostic metrics calculated using POD1 biomarker levels relative to postoperative sepsis status.

Marker	Optimal Cutoff (POD1)	Sensitivity	Specificity	PPV	NPV	Youden’s J
PCT (ng/mL)	1.10	0.955	0.875	0.830	0.969	0.830
CRP (mg/L)	315.00	0.705	0.529	0.516	0.717	0.234
IL-6 (pg/mL)	390.00	0.727	0.618	0.561	0.774	0.345

Procalcitonin demonstrated the highest diagnostic performance, with an AUC = 0.955, optimal cutoff = 1.10 ng/mL, sensitivity = 95.5%, specificity = 87.5%, and Youden’s J = 0.830. IL-6 showed moderate accuracy (AUC = 0.727, Youden’s J = 0.345**), while CRP exhibited comparatively lower discriminative ability (AUC = 0.705, Youden’s J = 0.234**). These findings indicate that PCT is the most reliable early biomarker for identifying postoperative sepsis following lung decortication.

ROC curve findings

Figure 2 illustrates the combined ROC curves for the three biomarkers. The PCT curve lies furthest from the diagonal reference line, confirming superior sensitivity and specificity across cutoff thresholds. The AUC for PCT was statistically significant (p < 0.001), whereas CRP and IL-6 did not differ significantly from the reference line (p > 0.05).

Summary of diagnostic metrics

Collectively, the results demonstrate that PCT is an early, highly sensitive, and specific indicator of postoperative sepsis. IL-6, though responsive to inflammatory stimuli, lacks adequate specificity due to overlap with non-infectious inflammation. CRP, being slower to normalize, is better suited for monitoring ongoing infection resolution rather than early detection.

## Discussion

This prospective observational study demonstrated that PCT is a significantly more reliable and earlier indicator of postoperative sepsis than CRP or IL-6 in patients undergoing lung decortication. All three biomarkers rose markedly after surgery; however, PCT showed a sharper peak on POD 1 and a steady decline thereafter, closely mirroring infection onset and resolution. In contrast, CRP and IL-6 exhibited delayed or nonspecific responses. ROC analysis confirmed PCT as the most accurate diagnostic marker (AUC = 0.955), followed by IL-6 (AUC = 0.727) and CRP (AUC = 0.705).

These findings are consistent with previous reports highlighting the diagnostic superiority of PCT for bacterial infection. Schuetz et al. and Becker et al. established that PCT rises within 6-12 hours after bacterial endotoxin exposure and correlates with sepsis severity, whereas CRP peaks later (24-48 hours) and lacks infection specificity [4,5]. The kinetic pattern observed in this study corroborates those observations. Meisner et al. and Christ-Crain et al. similarly demonstrated that PCT levels > 1 ng/mL strongly predict bacterial sepsis in postoperative or critical-care settings, whereas CRP remains persistently elevated even in sterile inflammation [10,11]. The present cutoff value of 1.10 ng/mL thus reinforces existing diagnostic thresholds.

IL-6 displayed moderate sensitivity but limited specificity, likely reflecting cytokine release triggered by surgical trauma rather than infection alone. Tanaka et al. and Castell et al. noted that IL-6 synthesis is promptly induced by tissue injury and hypoxia through NF-κB and STAT3 signaling, producing high postoperative variability. This explains the overlapping IL-6 levels observed between septic and non-septic groups [7,12]. While IL-6 serves as an early alarm for systemic inflammation, its non-specificity restricts its utility as an isolated biomarker of sepsis in thoracic surgery.

The physiological basis for PCT’s superior accuracy lies in its selective up-regulation by bacterial toxins and pro-inflammatory cytokines, such as IL-1β, TNF-α, and IL-6, while remaining suppressed during viral or non-infectious inflammation. Its short half-life enables real-time monitoring of disease progression and therapeutic response. In contrast, CRP, a hepatic acute-phase protein stimulated predominantly by IL-6, demonstrates slower kinetics and remains elevated beyond infection control. The combination of rapid induction and specific bacterial responsiveness makes PCT an ideal biomarker for early postoperative sepsis detection.

Clinically, the inclusion of PCT in postoperative surveillance could improve diagnostic precision and guide antibiotic stewardship. Its high sensitivity and specificity allow clinicians to differentiate between infectious and sterile inflammatory responses within the first 24 hours after surgery. A low PCT on POD 1 may safely exclude infection and reduce unnecessary antibiotic exposure, while persistently elevated values warrant aggressive sepsis work-up and timely intervention. These implications are consistent with prior trials demonstrating that PCT-guided therapy shortens antibiotic duration without compromising outcomes [13].

The strengths of this study include its prospective design, standardized biomarker sampling schedule, and simultaneous evaluation of three inflammatory mediators in a homogeneous thoracic cohort. Objective sepsis definitions and appropriate statistical modeling strengthen the reliability of results. Nonetheless, certain limitations merit consideration. Being a single-center study with a modest sample size, the findings may not be generalizable. Surgical heterogeneity, intraoperative blood loss, and comorbid factors were not stratified. Furthermore, culture-negative sepsis could have introduced misclassification bias, and long-term endpoints such as mortality or duration of hospitalization were not assessed.

Future multicenter investigations should validate these findings and establish procedure-specific PCT thresholds for thoracic surgery. Combining PCT with clinical scoring systems, such as sequential organ failure assessment (SOFA) or quick SOFA (qSOFA) [14], may enhance diagnostic accuracy. Integration of biomarker panels incorporating PCT with cytokines, presepsin, or high-mobility-group-box 1 protein may further refine early sepsis prediction.

## Conclusions

Procalcitonin demonstrated superior diagnostic accuracy compared with C-reactive protein and interleukin-6 for the early detection of postoperative sepsis following lung decortication. Among all biomarkers evaluated, procalcitonin rose rapidly within the first 24 hours after surgery and showed a strong correlation with infection onset and clinical recovery. Receiver-operating characteristic analysis confirmed procalcitonin as the most sensitive and specific predictor of postoperative sepsis, with an area under the curve of 0.955 and an optimal cut-off of 1.10 ng/mL.

Early measurement of procalcitonin can therefore serve as a valuable adjunct to clinical assessment, helping clinicians differentiate true sepsis from sterile postoperative inflammation. Incorporating procalcitonin monitoring into standard postoperative protocols may enable earlier diagnosis, guide antibiotic stewardship, and improve patient outcomes after thoracic surgery. Further multicenter studies with larger samples are recommended to validate these findings and establish standardized biomarker-based algorithms for sepsis surveillance in surgical patients.
